# Milk Exosome-Derived MicroRNA-2478 Suppresses Melanogenesis through the Akt-GSK3β Pathway

**DOI:** 10.3390/cells10112848

**Published:** 2021-10-22

**Authors:** In-Seon Bae, Sang Hoon Kim

**Affiliations:** Department of Biology, Kyung Hee University, Seoul 02447, Korea; since89_bis@naver.com

**Keywords:** melanogenesis, milk exosome, miR-2478, Rap1a

## Abstract

Exosomes participate in intercellular communication by transferring molecules from donor to recipient cells. Exosomes are found in various body fluids, including blood, urine, cerebrospinal fluid and milk. Milk exosomes contain many endogenous microRNA molecules. MicroRNAs are small noncoding RNAs and have important roles in biological processes. The specific biological functions of milk exosomes are not well understood. In this study, we investigated the effects of milk exosomes on melanin production in melanoma cells and melanocytes. We found that milk exosomes decreased melanin contents, tyrosinase activity and the expression of melanogenesis-related genes in melanoma cells and melanocytes. Bovine-specific miR-2478 in exosomes inhibited melanin production. We found that Rap1a is a direct target gene of miR-2478 in melanoma cells and melanocytes. MiR-2478 overexpression decreased Rap1a expression, which led to downregulated melanin production and expression of melanogenesis-related genes. Inhibition of Rap1a expression decreased melanogenesis through the Akt-GSK3β signal pathway. These results support the role of miR-2478 derived from milk exosomes as a regulator of melanogenesis through direct targeting of Rap1a. These results show that milk exosomes could be useful cosmeceutical ingredients to improve whitening.

## 1. Introduction

Cosmeceutical is a compound word combining cosmetic and pharmaceutical, and refers to cosmetics with functional ingredients that have medical effects [1]. In the past, skin was improved through dermatological procedures, but recently, interest in cosmeceuticals has been heightened due to consumers’ desire to obtain these effects through cosmetics. The cosmetic industry has also shown an increased interest in recent years in the use of novel cosmeceutical ingredients [2]. Functional cosmetics include products that help skin whitening by preventing the deposition of melanin on the skin.

Melanin, an important element that determines the color of skin and hair, protects skin from ultraviolet rays [3]. However, excessive formation of melanin causes uneven skin tone, e.g., spots and freckles [4]. Microphthalmia transcription factor (MITF) is a major regulator of melanocyte development. Tyrosinase (TYR) is the most important enzyme of melanin synthesis. The melanin synthesis process is induced by TYR activation via MITF, which is an important enzyme involved in the initial stage of the melanin synthesis process, and uses the L-form of tyrosine or DOPA as a substrate to form melanin [5].

MicroRNAs (miRs) are short noncoding RNAs that participate in many biological processes by controlling post-transcriptional gene expression [6]. Studies have also uncovered the control of melanin biosynthesis by microRNAs. For example, it has been reported that miR-143-5p targets the Tak1 gene and reduces MITF expression, thereby inhibiting melanin synthesis [7]. MiR-125b, a negative regulator of melanogenesis, has been demonstrated to regulate the pigmentation gene SH3BP4 [8], and miR-675 in exosomes secreted from keratinocytes inhibits melanogenesis by targeting MITF [9].

Exosomes are 30–200 nm sized membrane-bounded vesicles, and contain DNA, RNA and peptides [10]. The internal components of exosomes can safely transmit information to adjacent or remote cells without being degraded by enzymes in biological fluids, thus affecting the microenvironment around various cells [11]. Milk exosomes are milk-derived extracellular vesicles that are stable in human digestive processes [12]. The exosomes extracted from bovine milk contain not only several proteins such as casein and lactoglobulin but also microRNAs, which act as cargo [13,14,15]. Bovine milk contains immune-related microRNAs such as miR-15b, miR-27b, miR-34a and miR-10. These microRNAs are especially abundant in the colostrum [16].

Some studies have shown that components of milk inhibit melanogenesis in melanocytes; for example, proteins such as β-lactoglobulin and κ-casein in milk regulate melanogenesis [17,18]. The supernatant of *Lactobacillus helveticus* NS8-fermented milk has been found to inhibit UV damage and hyperpigmentation in the skin [19]. However, there is no report yet on whether microRNAs in bovine milk are involved in skin whitening, although 79 microRNAs exist in milk exosomes [13]. Therefore, in this study, we investigated whether milk exosomes containing microRNAs inhibit melanin synthesis in mouse and human melanoma cells and melanocytes.

## 2. Materials and Methods

### 2.1. Cell Culture

Mouse melanoma B16F10 cells were cultured in Dulbecco’s modified Eagle medium (DMEM, Hyclone, Logan, UT, USA). Human melanoma MNT-1 cells were grown in Minimum Essential medium (MEM, Hyclone). All the media were supplemented with 10% fetal bovine serum (FBS, Hyclone, USA) and 1% of a penicillin–streptomycin solution. Normal human epidermal melanocytes (NHEM, PromoCell, Heidelberg, Germany) at passage number 5 or 6 were maintained in a melanocyte growth medium (PromoCell). These cells were incubated at 37 °C in a humidified atmosphere condition containing 5% CO_2_.

### 2.2. Exosomes Purification

Commercial milk (1 mL) was centrifuged at 2000× *g* for 10 min, then the supernatant was centrifuged at 10,000× *g* for 10 min. The milk supernatant was passed through a 0.45 μm filter and then a 0.2 μm filter. The resulting solution (300 μL) was mixed with phosphate-buffered saline (PBS) and 300 μL of the Exoquick exosome precipitation solution (Systems Biosciences, Palo Alto, CA, USA) and incubated for 30 min. The mixture was centrifuged at 10,000× *g* for 30 min to obtain an exosome pellet, which was next resuspended in PBS. The size distribution of the exosomes was determined on a Zetasizer Nano ZS 90 (Malvern Instruments, Almelo, France). The exosomes were stored at −80 °C for further use.

### 2.3. Cryo-Electron Microscopy

A 3 μL aliquot of fresh exosomes suspension was adsorbed to a glow-discharged, perforated carbon-coated grid (2/2-3 C C-Flat; Protochips, Morrisville, NC, USA), which was then blotted for 3 s at 4 °C and plunge-frozen using a Vitrobot Mark IV (Thermo Fisher Scientific, Lafayette, CO, USA). The grids were stored in liquid nitrogen, then transferred to a Gatan 626 cryospecimen holder (Gatan, Pleasanton, CA, USA). The samples were imaged at a nominal magnification of 29,000× in a cryo-electron microscope (FEI Tecnai F20 TEM, FEI, Hillsboro, OR, USA), equipped with a standard field emission gun (s-FEG) and a K2 Summit camera (Gatan) at an accelerating voltage of 200 kV.

### 2.4. Cell Viability Assay (WST Assay)

Melanoma cells (B16F10, MNT-1) and human melanocytes (NHEM) were seeded at 4 × 10^3^ cells/well in 96-well plates and treated with milk exosomes (20 and 50 μg/mL) or a microRNA mimic (RNA double-strand oligonucleotides) for each experiment. At the indicated time points, cell viability was measured using the enhanced cell viability assay kit EZ-CyTox (Daeil Lab Service, Seoul, Korea) according to the manufacturer’s instructions. The absorbance was measured at 450 nm on a Vmax microplate spectrophotometer (Molecular Devices, San Jose, CA, USA).

### 2.5. Melanin Content Measurement

Melanoma cells and melanocytes were treated with milk exosomes (20 and 50 μg/mL) for 48 h. After washing twice with ice-cold PBS, the cells were centrifuged at 2500× *g* for 10 min. The cell pellets were resuspended at 90 °C for 30 min in 1 N NaOH containing 10% of dimethyl sulfoxide. Total melanin content was measured on the Vmax microplate spectrophotometer (Molecular Devices) at 405 nm.

### 2.6. Tyrosinase Activity Assay

Melanoma cells and melanocytes were incubated with milk exosomes for 48 h. The cells were washed twice with PBS and lysed with PBS containing 1% of Triton X-100 and 0.1 mM phenylmethanesulfonyl fluoride. After centrifugation, the supernatant was collected and transferred to a 96-well plate. After the addition of 0.1 M L-3,4-dihydroxyphenylalanine, samples were incubated at 37 °C for 1 h. The tyrosinase activity was measured on the basis of the absorbance at 475 nm using the microplate spectrophotometer (Molecular Devices).

### 2.7. Quantitative rReverse-Transcription Polymerase Chain Reaction (qRT-PCR)

For qRT-PCR analysis of the mRNA, the extracted total RNA was converted to cDNA with MML-V reverse transcriptase (Promega, Madison, WI, USA) according to the manufacturer’s instructions. The cDNA of TYR, MITF, Rap1a and β-actin was quantitatively detected by a Rotor-Gene Q PCR instrument (Qiagen, Hilden, Germany) using the SYBR Green PCR Master mix (Bioline, London, UK). PCR was carried out under the following conditions: 2 min at 95 °C, then 40 cycles of 4 s at 95 °C, 10 s at 60 °C and 15 s at 72 °C. The sequences of the PCR primers were as follows: forward, 5′-CAGGAACCGAGCAATTTACAGC-3′ and reverse, 5′-TGTTCTTTGCCAACTACCCGT-3′ for Rap1a; forward, 5-GTGACGTTGACATCCGTAAAG-3′ and reverse, 5′-GCCGGACTCATCGTACTCC-3′ for β-actin. The amount of target mRNA was normalized to β-actin mRNA as the internal control.

For qRT-PCR analysis of microRNA, total RNA was isolated using TRIzol (Invitrogen, Waltham, MA, USA). cDNA was synthesized from the microRNA using the miScript Reverse Transcription kit (Qiagen). The expression levels were quantified with the miScript SYBR Green kit (Qiagen). Specific primers were purchased from Qiagen. The reaction was performed via the following thermal cycling program: 94 °C for 15 min, followed by 40 cycles of 94 °C for 15 s, 55 °C for 30 s and 70 °C for 20 s. The relative expression was calculated by the 2^−^^ΔΔCt^ method.

### 2.8. Transfection and Luciferase Reporter Assay

The small interfering RNA (siRNA) oligonucleotides against mouse Rap1a and the microRNA mimic were purchased from GenePharma (Shanghai, China). B16F10 and MNT-1 cells were transfected with the siRNA oligonucleotides or the microRNA mimic using Lipofectamine 2000 (Invitrogen) according to the manufacturer’s instructions. For the assessment of luciferase activity, Cos7 cells were cotransfected with the pGL3-Rap1a plasmid and either a negative control or the miR-2478 mimic. The miR-2478 binding site in the Rap1a 3′ untranslated region (3′UTR) in the pGL3-control plasmid was mutated by means of the QuickChange Site-Directed Mutagenesis kit (Stratagene, La Jolla, USA). At 48 h post-transfection, luciferase activities were evaluated using the dual luciferase kit (Promega) on a Glomax 20/20 luminometer (Turner Biosystems, Sunnyvale, CA, USA).

### 2.9. Western Blotting

Cells were lysed with a RIPA cell lysis buffer (50 mM Tris-HCl, 150 mM NaCl, 1% NP-40, 0.1% SDS, a protease inhibitor cocktail, 50 mM NaF and 0.2 M NA_3_VO_4_). Proteins were separated by a 10% sodium dodecyl sulfate-polyacrylamide gel, and transferred onto a nitrocellulose membrane (Whatman International Ltd., Kent, UK). After blockage with Tris-buffered saline containing 0.1% Tween 20 and 5% skim milk, the membranes were incubated with anti-MITF (Abcam, Cambridge, MA, USA), anti-TYR (Abcam), anti-Rap1a (Novus, Centennial, CO, USA), anti-Akt (Cell Signaling Technology, Danvers, MA, USA), anti-phospho-Akt (Cell Signaling Technology), anti-GSK3β (Cell Signaling Technology), anti-phospho-GSK3β (Cell Signaling Technology) and anti-actin (Sigma-Aldrich, Burlington, MA, USA) antibodies. After washing, the membranes were incubated with horseradish peroxidase-conjugated secondary antibodies. The target protein’s signals were developed with the enhanced chemiluminescence kit (Santa Cruz Biotechnology, Inc., Dallas, TX, USA).

### 2.10. Human Skin Tissues

Epidermal equivalents containing melanocytes (MelanoDerm, MEL-300B) were obtained from MatTek (Ashland, MA, USA) and were maintained in EPI-100-NMM-113-medium. Milk exosomes were applied to MelanoDerm tissues on Days 1, 3, 7, 9 and 12. PBS was used as the negative control. Tissues were photographed on Days 3, 7, 9, 12 and 14. Pigmentation was measured by comparing the changes in the L value (Adobe Photoshop CC 2015). Melanin contents in human skin tissue were measured on Day 14 by a Solvable melanin assay. Briefly, the MelanoDerm tissues were solubilized using Solvable (Perkin Elmer, Waltham, MA, USA) and incubated overnight at 60 °C. Samples were vortexed and then centrifuged at 16,500× *g* for 5 min. The supernatant was transferred into the wells of a 96-well plate and read at 490 nm by a spectrophotometer.

### 2.11. Histological Analysis

MelanoDerm tissues were fixed in 10% formalin, embedded in paraffin, cut to a thickness of 4 μm and subjected to with hematoxylin and eosin staining (H&E) and Fontana–Masson silver staining (F&M, Abcam). For H&E, paraffin sections were deparaffinized and then hydrated in ethanol. Next, sections were stained with hematoxylin for 5 min and eosin for 30 s. After staining, samples were washed in running tap water. For F&M, paraffin sections were incubated with an ammoniacal silver solution for 1 h at 60 °C, followed by washing 3 times in distilled water. Samples were incubated with a 0.2% gold chloride solution for 1 min and immediately rinsed with running water for 2 min. Samples were incubated with a nuclear fast red solution for 5 min and rinsed with running water for 1 min. The stained samples were covered with a mounting solution and examined under a microscope (CK40, Olympus, Tokyo, Japan).

### 2.12. Statistical Analysis

Experiments were performed at least 3 times and the values represent the mean ± standard deviation. Statistical significance was analyzed by Student’s *t*-test. The statistical analysis was performed in GraphPad Prism 5 (GraphPad Software, La Jolla, CA, USA).

## 3. Results

### 3.1. Characterization of the Exosomes Isolated from Milk

**Figure 1:**
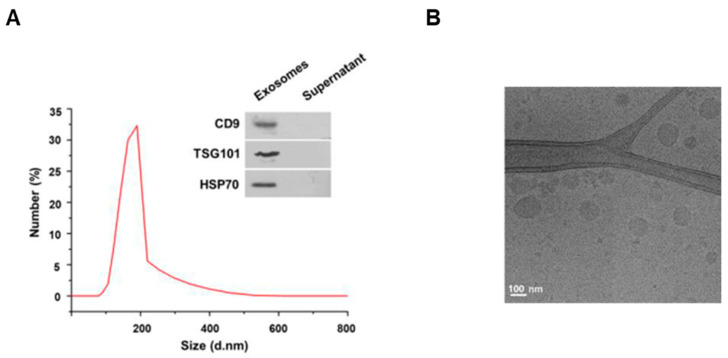
Characterization of milk exosomes. (**A**) The size distributions of milk exosomes were determined by dynamic light scattering; the inset shows Western blotting of the exosome marker genes CD9, TSG101 and HSP70 in the milk exosome pellet and supernatant after centrifugation during the exosome isolation procedure. (**B**) An image of milk exosomes captured by cryo-electron microscopy.

To investigate the characteristics of the exosomes extracted from cows’ milk, we first measured the size of the exosomes by dynamic light scattering. As a result, the exosomes extracted from milk were found to have a diameter of 80–190 nm (Figure 1A). For milk exosome purification, the Exoquick–milk supernatant mixture was centrifuged to obtain a milk exosome pellet. The expression of CD9, TSG101 and HSP70 as exosomal markers was detected in pellets, but these proteins were not present in the supernatant (Figure 1A). In addition, the spherical morphology and size of the exosomes were confirmed by cryo-electron microscopy (Figure 1B). Exosomes were found to have a round shape of up to 200 nm in diameter. Next, we investigated whether the exosomes extracted from milk were taken up by cells. Mouse B16F10 cells were incubated with milk exosomes labeled with the lipophilic fluorescent dye PKH26. As a result, we observed that the labeled exosomes were present inside the cells (Figure S1).

### 3.2. Tyrosinase Activity and Melanin Production Suppressed by Milk Exosomes in B16F10 Cells

**Figure 2:**
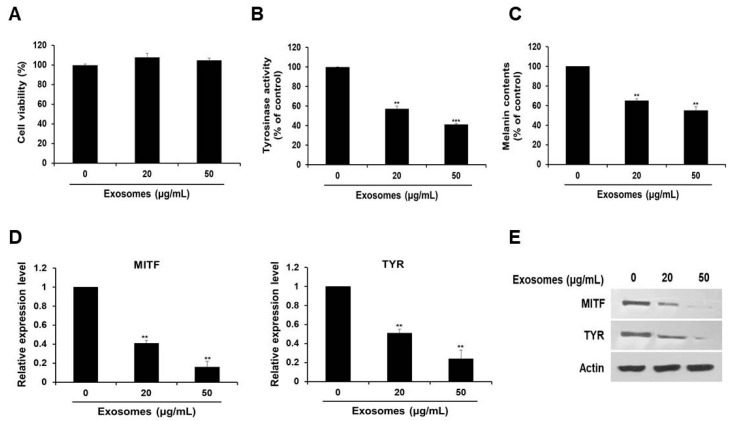
Milk exosomes reduce melanogenesis in mouse B16F10 cells. Cells were cultured with 20 or 50 μg/mL of exosomes for 48 h. (**A**) Cell viability was measured by a WST assay. (**B**) Tyrosinase activity was assessed after treatment with milk exosomes; n = 3, ** *p* < 0.01, *** *p* < 0.001. (**C**) Melanin contents were determined in the cells exposed to milk exosomes; n = 3, ** *p* < 0.01. (**D**) MITF and TYR mRNA levels in milk exosome-treated cells were measured by qRT-PCR; n = 3, ** *p* < 0.01. (**E**) Levels of the proteins MITF and TYR in cells treated with milk exosomes were examined by Western blotting.

Next, we examined the effect of the extracted exosomes on cell viability. As depicted in Figure 2A, we revealed that there was no significant difference in cell viability, regardless of the exosome concentration.

To investigate the possible skin cell-whitening effect of milk exosomes, cells were treated with milk exosomes. We found that tyrosinase activity in cells exposed to milk exosomes at concentrations of 20 or 50 μg/mL diminished by 43% and 59%, respectively (Figure 2B). Because the reduction of tyrosinase enzyme activity can inhibit melanin biosynthesis, melanin contents were measured after treatment with milk exosomes. We noted inhibition of melanin synthesis in cells exposed to milk exosomes (20 or 50 μg/mL) by 45% and 55%, respectively (Figure 2C). Furthermore, the expression of MITF mRNA decreased as the concentration of milk exosomes increased. The level of MITF mRNA decreased by 59% in the cells treated with 20 μg/mL exosomes and by 84% in the cells treated with 50 μg/mL exosomes, respectively (Figure 2D). Tyrosinase (TYR) mRNA expression also diminished by 49% and 76% in the cells exposed to these concentrations of exosomes (Figure 2D). MITF and tyrosinase protein levels also decreased as the concentration of milk exosomes increased (Figure 2E). These results suggest that milk exosomes inhibit melanogenesis in mouse B16F10 cells.

### 3.3. Enrichment of Bovine-Specific miR-2478 in Milk Exosomes

**Figure 3:**
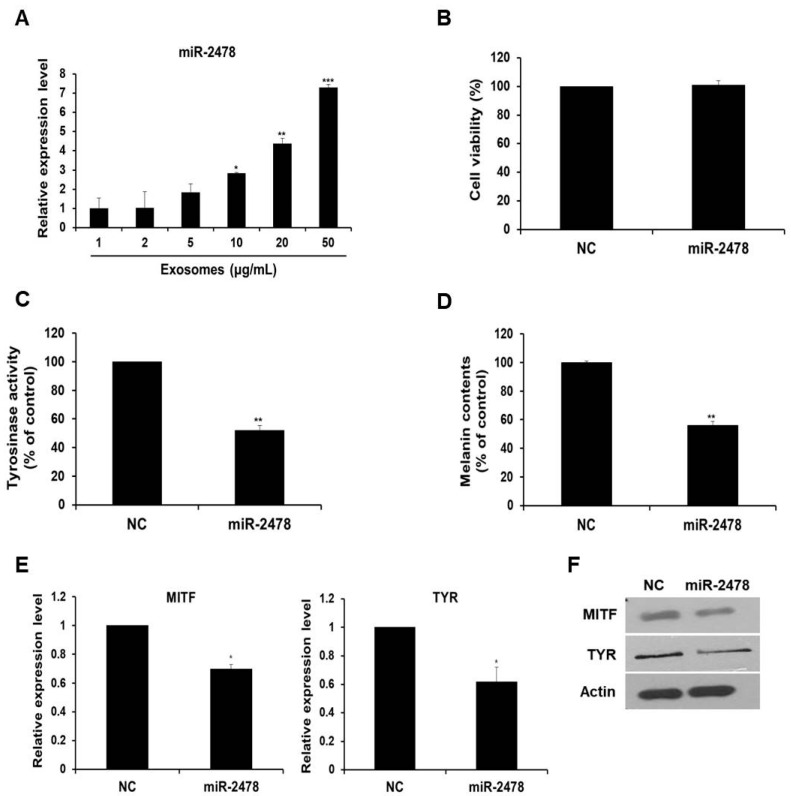
miR-2478 inhibits melanogenesis. (**A**) MiR-2478 expression levels in cells treated with milk exosomes were measured by qRT-PCR; n = 3, * *p* < 0.05, ** *p* < 0.01, *** *p* < 0.001. (**B**) B16F10 cells were transfected with the miR-2478 mimic or negative control (NC). At 48 h post-transfection, the cell viability assay (WST assay) was performed. (**C**,**D**) Tyrosinase activity and melanin contents were measured in the cells transfected with the NC or the miR-2478 mimic; n = 3, ** *p* < 0.01. (**E**,**F**) At 48 h post-transfection of the miR-2478 mimic or NC, the expression of MITF and TYR in the cells was measured by qRT-PCR and Western blotting; n = 3, * *p* < 0.05.

Next, we determined whether milk exosome-derived microRNA inhibited the expression of melanogenesis-related genes. An experiment on the microRNA profile of bovine milk exosomes showed that bovine-specific miR-2478 was the most abundant microRNA in milk exosomes [13]. Based on this work, we selected miR-2478 and conducted a study on whether miR-2478 plays a role in melanogenesis. First, we tested whether the miR-2478 concentration increased as the concentration of milk exosomes increased. When milk exosomes were incubated with B16F10 cells, it was confirmed that miR-2478 levels increased inside cells as the milk exosome concentration increased (Figure 3A). There was no difference in cell viability between control and miR-2478-transfected cells (Figure 3B). Next, a miR-2478 mimic was transfected into B16F10 cells to investigate the possible whitening effect of miR-2478. In cells transfected with the miR-2478 mimic, tyrosinase activity decreased by 48% (Figure 3C) and melanin contents diminished by 44% (Figure 3D). In addition, MITF and TYR mRNA expression in the cells transfected with the miR-2478 mimic decreased by 30% and 38%, respectively, compared with the control (Figure 3E). Levels of the proteins MITF and TYR also decreased in the cells transfected with the miR-2478 mimic (Figure 3F). These data indicate that the miR-2478 mimic inhibited tyrosinase activity and melanin synthesis, thereby exerting a whitening effect.

### 3.4. Bovine-Specific miR-2478 from Milk Exosomes Suppresses Melanogenesis

**Figure 4:**
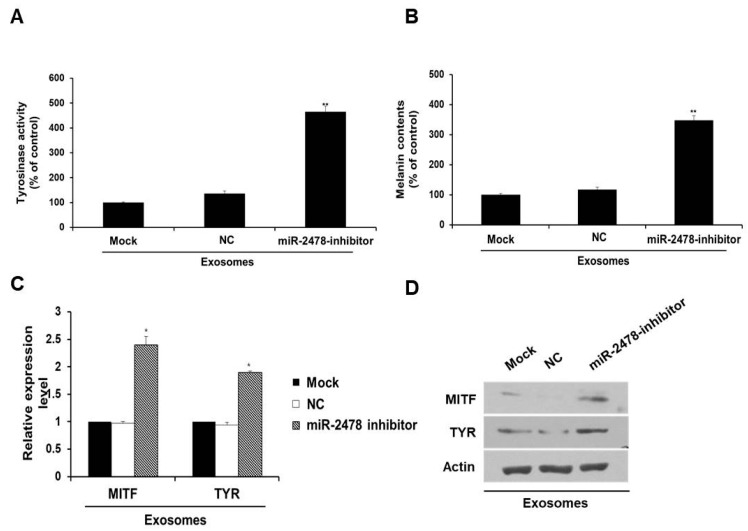
Silencing of miR-2478 recovers melanogenesis in milk exosome-treated cells. (**A**,**B**) Tyrosinase activity and melanin contents in the cells treated with the negative control (NC) or the miR-2478 inhibitor were analyzed in the presence of 50 μg/mL of exosomes; n = 3, ** *p* < 0.01. (**C**,**D**) Expression of MITF and TYR was measured in NC or miR-2478 inhibitor-transfected cells with milk exosomes, as assessed by qRT-PCR and Western blotting; n = 3, * *p* < 0.05.

In order to determine whether miR-2478 derived from milk exosomes directly affects melanogenesis, the miR-2478 inhibitor was transfected into the cells exposed to milk exosomes (50 μg/mL). In the presence of milk exosomes, B16F10 cells transfected with the miR-2478 inhibitor showed significantly higher tyrosinase activity compared with that in the control (Figure 4A). Melanin contents were also higher in miR-2478 inhibitor-treated cells than in control cells (Figure 4B). MITF mRNA expression was –2.5-fold higher in miR-2478 inhibitor-treated cells than in control cells, and TYR mRNA expression was –2-fold higher when miR-2478 was inhibited (Figure 4C). The levels of MITF and TYR proteins also recovered in the cells exposed to milk exosomes after these cells were transfected with the miR-2478 inhibitor (Figure 4D). These results suggested that miR-2478 derived from milk exosomes directly suppressed melanogenesis in mouse melanoma cells.

### 3.5. Rap1a Is Directly Targeted by miR-2478

**Figure 5:**
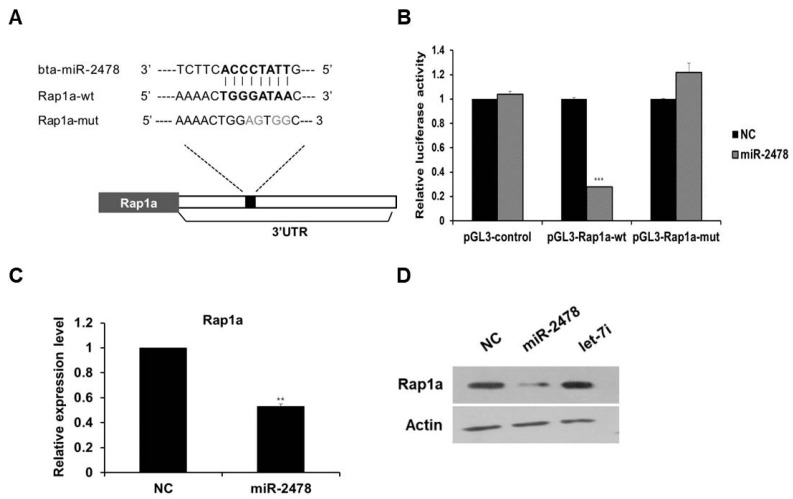
Rap1a is targeted by miR-2478. (**A**) Sequence alignment of a putative binding site for miR-2478 in the 3′UTR of Rap1a mRNA. The putative binding site in the Rap1a 3′UTR region was then mutated. (**B**) Cos7 cells were cotransfected with either the miR-2478 mimic or a negative control (NC) for 48 h and either a wild-type 3′UTR reporter plasmids(pGL3-Rap1a-wt) or a mutant 3′UTR plasmid (pGL3-Rap1a-mut). Luciferase activity was assayed at 48 h post-transfection; n = 3, *** *p* < 0.001. (**C**,**D**) Levels of Rap1a expression in B16F10 cells transfected with either the miR-2478 mimic or NC were measured by qRT-PCR and Western blotting; n = 3, ** *p* < 0.01.

To investigate the mechanism of miR-2478-mediated suppression of melanogenesis in cells, we first identified the target gene of miR-2478. As a result of analysis in the miRNA–target prediction algorithm program TargetScan (http://www.targetscan.org/vert_72/, accessed on 20 October 2021), Rap1a was selected as a target gene of miR-2478, and this miR-2478 target turned out to be conserved between mice and humans. We found the potential miR-2478-binding site in the 3’-UTR region of Rap1a, and a mutated plasmid was constructed (Figure 5A). The luciferase reporter assay showed that luciferase activity decreased by around 75% in the cells transfected with the miR-2478 mimic as compared with the control (Figure 5B). In contrast, the luciferase activity of the cells transiently transfected with the plasmid carrying the mutated miR-2478-binding site in Rap1a did not differ from the controls’ luciferase activity. In addition, Rap1a mRNA expression decreased in the cells overexpressing miR-2478 (Figure 5C). Levels of the Rap1a protein in miR-2478-transfected cells decreased compared with the control, whereas there was no change in Rap1a protein expression in the cells transfected with let-7i, which did not respond to miR-2478 (Figure 5D). These results indicate that Rap1a is a direct target of miR-2478 in mouse melanoma cells.

### 3.6. Rap1a Promotes Melanogenesis

**Figure 6:**
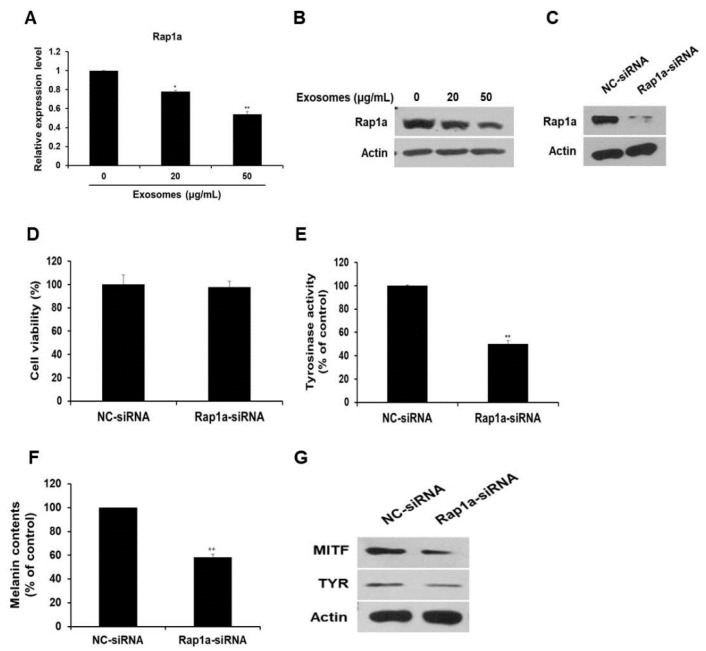
Silencing of Rap1a suppresses melanogenesis. (**A**,**B**) Levels of Rap1a mRNA and protein in B16F10 cells treated with milk exosomes were examined by qRT-PCR and Western blotting; n = 3, * *p* < 0.05, ** *p* < 0.01. (**C**) Levels of Rap1a protein were determined by Western blotting in negative control siRNA (NC-siRNA) or Rap1a siRNA-treated cells. (**D**) B16F10 cells were transfected with NC-siRNA or Rap1a-siRNA. A cell viability assay (WST assay) was performed at 48 h post-transfection. (**E**,**F**) Tyrosinase activity and melanin contents were assayed in the cells transfected with NC-siRNA or Rap1a-siRNA; n = 3, ** *p* < 0.01. (**G**) Expression of the proteins MITF and TYR was measured in Rap1a siRNA-treated cells by Western blotting.

Next, the expression of the miR-2478 target gene Rap1a was examined in milk exosome-treated cells. Expression of Rap1a mRNA decreased by 22% and 46% in B16F10 cells exposed to 20 or 50 μg/mL of milk exosomes, respectively (Figure 6A). Expression of the Rap1a protein also decreased in a milk exosome concentration-dependent manner (Figure 6B). Because the function of Rap1a in melanoma cells has not been elucidated yet, we investigated this function in relation to whitening. First, siRNA was prepared to inhibit Rap1a expression. Figure 6C illustrates the reduction in intracellular Rap1a protein expression by siRNA oligo. The inhibition of Rap1a expression in the cells did not affect cell viability (Figure 6D). The cells transfected with the Rap1a siRNA oligo contained around 50% lower tyrosinase activity than the control did (Figure 6E). Melanin contents diminished by around 42% in the Rap1a siRNA-treated cells (Figure 6F). In addition, the amount of MITF and TYR proteins decreased in Rap1a-deficient cells compared with the control (Figure 6G). These results suggest that Rap1a promotes melanogenesis in mouse melanoma cells.

### 3.7. Milk Exosomes Suppress Melanogenesis through the Akt-GSK3β Pathway

**Figure 7:**
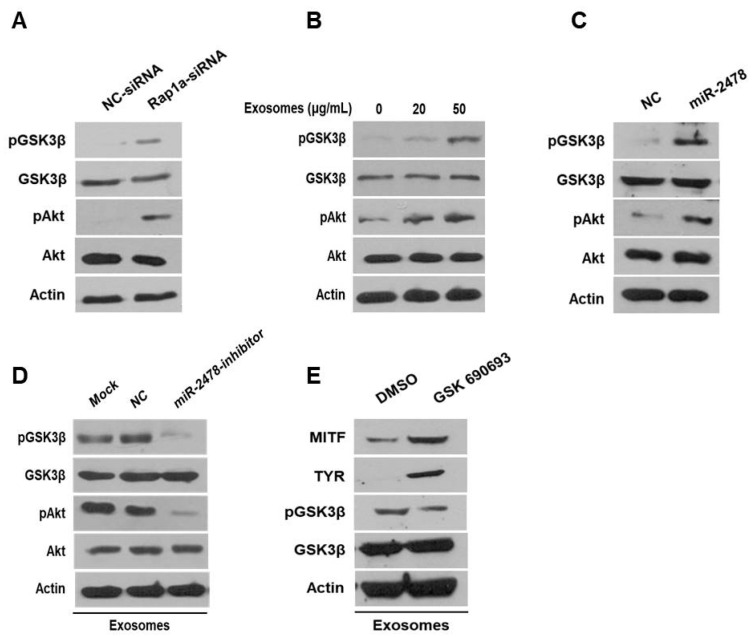
The Akt-GSK3β signaling pathway is affected by milk exosomes. (**A**) Protein levels of pAkt, total Akt, pGSK3β and total GSK3β in B16F10 cells transfected with NC-siRNA or Rap1a-siRNA were measured by Western blotting. (**B**) Akt and GSK3β protein levels were analyzed in cells treated with 20 or 50 μg/mL of milk exosomes. (**C**) After the cells were transfected with a negative control (NC) or a miR-2478 mimic, Akt and GSK3β protein levels were assayed by Western blotting. (**D**) In the presence of 50 μg/mL of exosomes, cells were transfected with the NC or miR-2478 inhibitor. (**E**) Cells exposed to 50 μg/mL of exosomes were treated with the Akt inhibitor GSK 690693. The levels of MITF, TYR and GSK3β in B16F10 were analyzed by Western blot analysis.

Next, we examined how milk exosomes regulate melanogenesis to identify the relevant affected pathway in melanoma cells. Although it is not known whether Rap1a directly controls melanogenesis, it has been reported that Rap1a inhibits Akt phosphorylation in cancer cells [20]. On the basis of this information, we investigated whether Rap1a modulates the Akt pathway to promote melanogenesis. In the Akt-related melanogenesis pathway, activated Akt induces the phosphorylation of Gsk3β at Ser9 and thus inactivates it; this change inhibits he transcriptional activity of MITF and downregulates tyrosinase [21,22]. In the present study, we evaluated the expression of Akt and GSK3β in cells treated with Rap1a siRNA. In these cells, activation of Akt phosphorylation increased and the amount of pGSK3β also increased (Figure 7A). GSK3β was inactivated by activation of Akt in a concentration-dependent manner in the cells treated with 20 or 50 μg/mL of milk exosomes (Figure 7B). The levels of the proteins Akt and GSK3β in the cells treated with the miR-2478 mimic were consistent with the above results (Figure 7C). Next, we tested whether miR-2478 from milk exosomes affects the Akt-GSK3β pathway. In the presence of milk exosomes, the phosphorylation of the proteins Akt and GSK3β was lower in the cells treated with the miR-2478 inhibitor (Figure 7D). In addition, treatment with the AKT inhibitor GSK690693 increased the expression of genes associated with melanogenesis in milk exosome-treated cells. (Figure 7E). These findings meant that miR-2478 in milk exosomes downregulates Rap1a and suppresses melanogenesis through the Akt-GSK3β pathway.

### 3.8. Milk Exosomes Inhibited Melanogenesis in Human Melanoma Cells, Melanocytes and MelanoDerm Tissue

**Figure 8:**
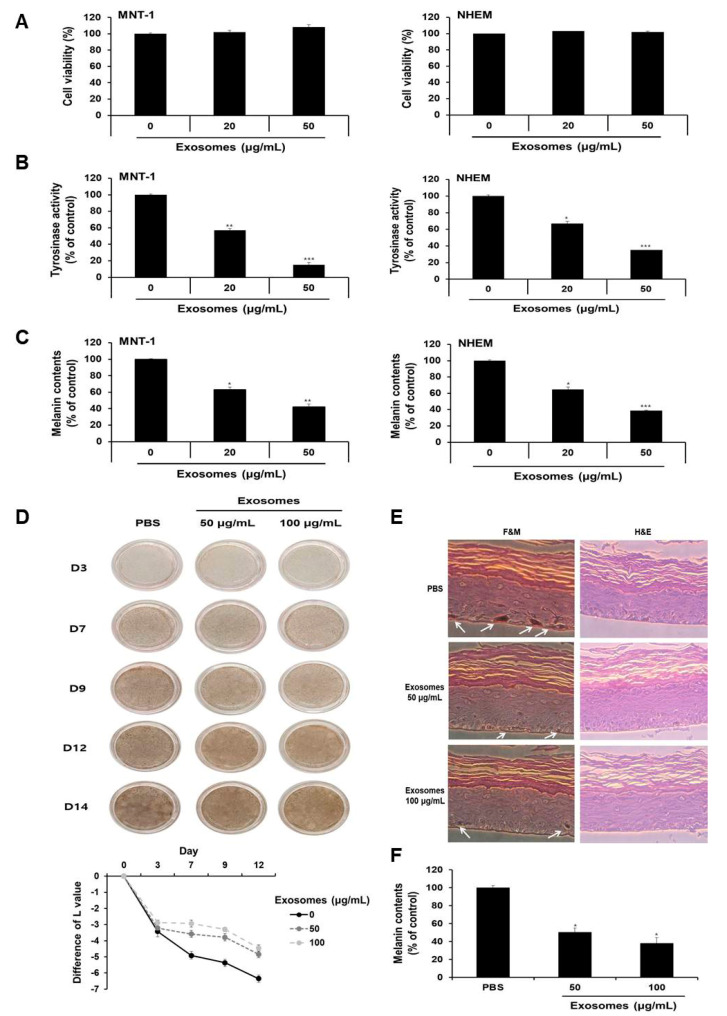
Milk exosomes suppress melanogenesis in human melanoma cells and melanocytes. (**A**) MNT-1 human melanoma cells and NHEM human melanocytes were exposed to 20 or 50 μg/mL of milk exosomes for 48 h, and cell viability was evaluated by a WST assay. (**B**,**C**) Tyrosinase activity and melanin contents in the cells treated with milk exosomes were measured; n = 3, ** *p* < 0.01, *** *p* < 0.001. (**D**) Human skin tissues (MelanoDerm) were treated with PBS as a control, or 50 or 100 μg/mL of exosomes, and then photographed. The level of pigmentation in the skin was measured by the L value. (**E**) Paraffin-embedded tissue sections were stained using hematoxylin and eosin (H&E). The melanin pigment of MelanoDerm was visualized by Fontana–Masson staining (F&M). (**F**) Melanin contents were determined in the MelanoDerm tissues exposed to milk exosomes; n = 3, * *p* < 0.05.

Given that milk exosomes inhibited melanogenesis in mouse cells, we next determined whether milk exosomes were equally effective in human cells. First, the viability of MNT-1 human melanoma cells and NHEM human melanocytes were examined after incubation with 20 or 50 μg/mL of milk exosomes. As presented in Figure 8A, there was no difference in viability. Tyrosinase activity was inhibited by 43% and 85%, respectively, in MNT-1 cells exposed to 20 μg/mL or 50 μg/mL of milk exosomes (Figure 8B). NHEM also reduced by 34% and 66%, respectively, through treatment with 20 μg/mL or 50 μg/mL of milk exosomes (Figure 8B). Furthermore, melanin contents were measured in MNT-1 cells and NHEM exposed to milk exosomes. As a result, melanin synthesis in MNT-1 cells exposed to 20 μg/mL or 50 μg/mL of milk exosomes was inhibited by 37% and 58%, respectively, and in NHEM cells, the milk exosome treatment hindered melanin synthesis by 36% and 62%, respectively (Figure 8C). In addition, the skin-lightening effect of milk exosomes was further confirmed using human skin tissues. After treatment with milk exosomes in human tissues, relative whitening effects were observed on Days 3, 9, 12 and 14. As a result, we found that the skin color in milk exosome-exposed tissues appeared to be brighter than that in the control tissues (Figure 8D). The degree of pigmentation was calculated as the L value, which is a value representing brightness. Pigmentation progressed more slowly in milk exosome-treated skin tissues than in control skin tissues (Figure 8D). The experimental group treated with milk exosomes showed a smaller melanin particle size and a low amount of melanin pigment compared with the control group (Figure 8E). As a result of measuring the melanin contents after 14 days of milk exosome treatment in human tissue, we confirmed that milk exosomes inhibited melanin synthesis in human tissues (Figure 8F). Therefore, we concluded that milk exosomes inhibit melanogenesis in human cells and tissues.

### 3.9. miR-2478 Inhibited Melanogenesis in Human MNT-1 Melanoma Cells and NHEM

**Figure 9:**
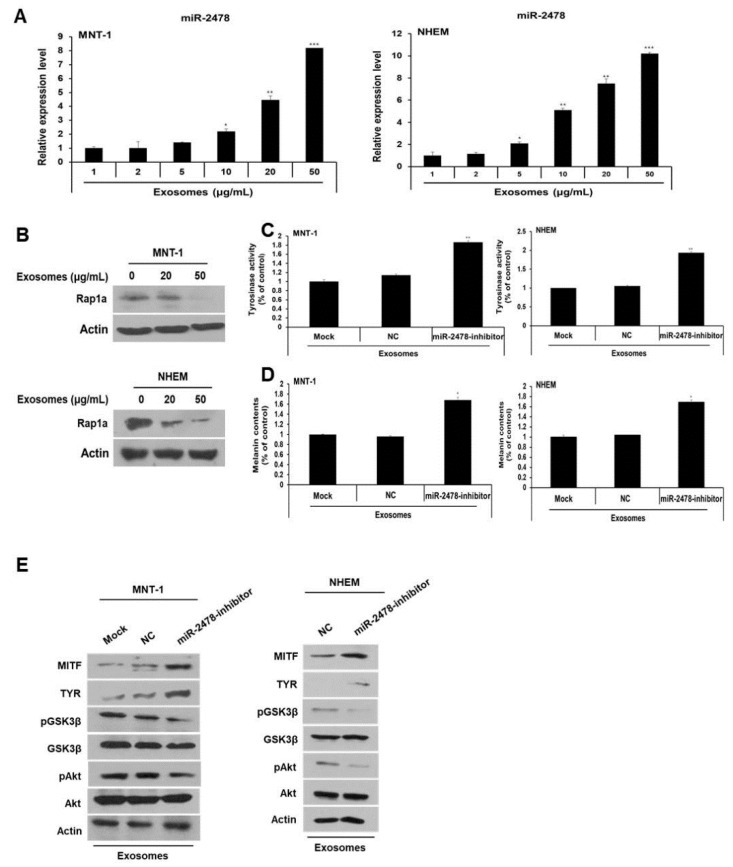
miR-2478 inhibits melanogenesis in human melanoma cells and melanocytes. (**A**) MiR-2478 expression levels in cells treated with milk exosomes were measured by qRT-PCR; n = 3, * *p* < 0.05, ** *p* < 0.01, *** *p* < 0.001. (**B**) The Rap1a protein level in milk exosome-treated cells was assessed by Western blotting. (**C**,**D**) Tyrosinase activity and melanin contents were examined in the negative control (NC) or miR-2478 inhibitor-treated cells with 50 μg/mL of exosomes; n = 3, * *p* < 0.05. ** *p* < 0.01. (**E**) Protein levels of MITF, TYR, pAkt, total Akt, pGSK3β and total GSK3β in NC or miR-2478 inhibitor-transfected cells exposed to milk exosomes were measured by Western blotting.

Next, we investigated the miR-2478 level in MNT-1 cells and NHEM exposed to milk exosomes. When these cells were incubated with milk exosomes at various concentrations, it was confirmed that miR-2478 levels increased in these cells as the milk exosome concentration increased (Figure 9A). Furthermore, when the cells were exposed to milk exosomes, levels of the Rap1a protein decreased in a milk exosome concentration-dependent manner (Figure 9B). When the miR-2478 inhibitor was transfected into the cells treated with milk exosomes, tyrosinase activity and melanin contents recovered in these cells (Figure 9C and 9D). Inhibition of miR-2478 expression in human cells exposed to exosomes reduced Akt and GSK3β phosphorylation (Figure 9E). Expression of the proteins MITF and TYR was also increased in these cells (Figure 9E). Thus, we confirmed that miR-2478 inhibits melanogenesis in human cells.

## 4. Discussion

Studies on exosomes regulating melanogenesis have reported that exosomes derived from keratinocytes have a whitening effect. Melanocytes and the surrounding keratinocytes are functionally connected to form melanin units in the skin epidermis [23,24]. Keratinocyte-derived soluble factors regulate the melanogenesis of neighboring melanocytes. Exosomes secreted from normal keratinocytes promote melanogenesis by upregulating the expression of melanogenesis-related proteins [23,24,25]. In addition, in a whitening study using exosomes extracted from cells, it was recently reported that exosomes extracted from human adipose tissue-derived stem/stromal cells showed skin brightening efficacy in mouse melanoma cells [26]. There was also a report that exosomes extracted from plants have a whitening effect. The extracellular vesicles extracted from *D. morbifera* plant leaves and stems exhibited anti-melanogenic effects on mouse melanoma cells and healthy human skin [27]. In our study, the whitening effect of milk-derived exosomes was investigated.

Compared with exosomes extracted from blood plasma or culture fluid, exosomes derived from milk have the advantages of not only having large amounts of exosomes that can be extracted per unit of volume but also a low price [26,27]. Because milk exosomes have a chemical composition similar to that of cell membranes, they exhibit lower immunogenicity and cytotoxicity, and have high biocompatibility [28,29]. Recently, when milk exosomes were fed to DSS (dextran sulfate sodium salt)-induced colitis model mice, the expression of the proinflammatory cytokines IL-6 and TNFα decreased compared with the control group, thereby reducing inflammation and improving necrotizing enteritis [30,31]. In addition, the ingestion of milk exosomes in a mouse model of osteoporosis disease improved arthritis by increasing bone density and reducing proinflammatory cytokines [32,33]. So far, the effects of milk exosomes have been focused on immunological phenomena that reduce proinflammatory cytokines, and on bone development, such as alleviation of enteritis and improvement of osteoporosis. In this study, besides the method of taking milk exosomes, it was confirmed that when applied to the skin, it was effective in skin whitening. This indicates that it can be applied for the treatment of various diseases by enhancing physiological activity using milk exosomes in the future.

Although this study suggested that milk exosomes inhibit melanogenesis, it is already known that whey proteins such as casein, β-lactoglobulin and α-lactalbumin have a whitening effect. β-lactoglobulin decreases tyrosinase activity in human melanocytes [18], and k-casein inhibits melanogenesis in mouse B16 melanoma cells [17]. Interestingly, many of these components are also present in milk exosomes. According to the proteome results of milk exosomes, casein, β-lactoglobulin and α-lactalbumin are distributed in the exosomes [15,34,35]. Milk exosomes contain microRNAs as well as proteins. As a result of a miRNA microarray using milk exosomes by Izumi and colleagues, miR-2478, miR-1777b, miR-1777a, let-7b and miR-1224 were highly distributed in the milk exosomes [13]. In this study, with the exception of miR-2478, among these microRNAs, miR-1777b, miR-1777a, let-7b and miR-1224 showed no difference in tyrosinase activity from the control group (Figure S2). miR-2478 was most abundant in milk exosomes and decreased tyrosinase activity. However, the expression of whitening-related MITF and TYR genes decreased more in the cells treated with milk exosomes than in the cells treated with the miR-2478 mimic alone. These results indicate that several components, including microRNAs in milk exosomes, are involved in the whitening action.

Most of the microRNAs in milk exosomes exert biological functions by inhibiting the expression of target genes in the recipient cells. In this study, miR-2478 also inhibited melanin synthesis by targeting Rap1a as a target gene. Although they were not milk-derived microRNAs, microRNAs that control the expression of whitening-related genes have already been reported. When the human miR-27a-3p mimic was applied to mouse melanocytes, it inhibited Wnt3a expression and thereby inhibited melanin synthesis [36], and ectopic miR-218 inhibited TYR activity by reducing MITF expression in murine melan-a melanocytes [37]. miR-203 promotes melanogenesis through the CREB1/MITF/Rab27a pathway by targeting Kif5b in melanoma [38]. There are 79 microRNAs in milk exosomes derived from cows [13]. Therefore, further research on the physiological activity of milk exosomes is needed.

## 5. Conclusions

**Figure 10:**
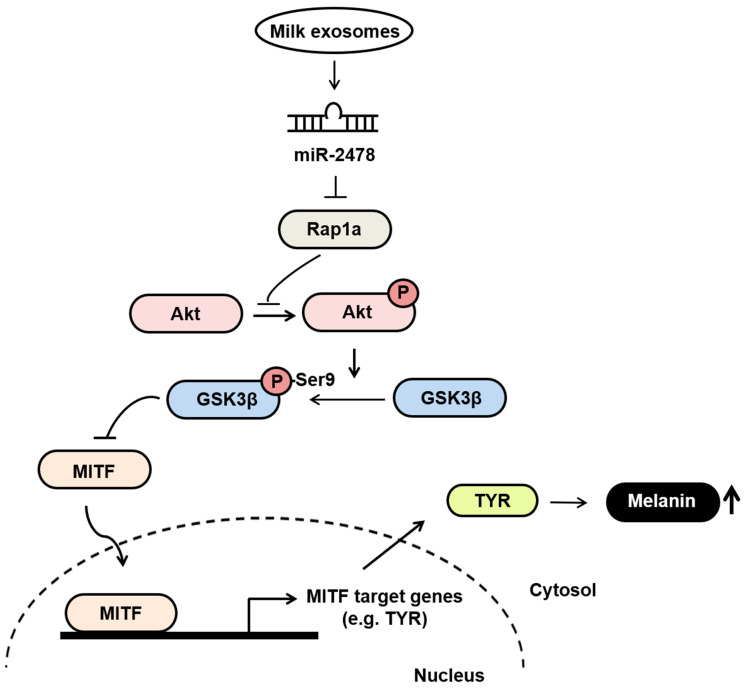
The proposed model explaining how milk exosomes inhibit melanin production in melanoma cells. In the absence of milk exosomes, Rap1a maintains the active form of GSK3β by inhibiting Akt phosphorylation. The dephosphorylation of GSK3β upregulates the melanogenesis-related protein MITF, which promotes melanin production by driving the expression of tyrosinase. However, in the presence of milk exosomes, miR-2478-carrying milk exosomes inhibit Rap1a expression in melanoma cells, and thus promote the activation of its downstream mediator Akt (via phosphorylation), resulting in GSK3β phosphorylation at Ser9; this change inactivates GSK3β and represses the expression of melanogenesis-related genes such as MITF and TYR, thereby inhibiting melanin production.

Our findings suggest that milk exosomes are candidates for anti-melanogenic agents that decrease pigmentation by inhibiting the expression of melanin-related genes (Figure 10). In melanoma cells and melanocytes, milk exosomes showed TYR inhibition and melanin content reduction compared with the negative control. In addition, these effects were mediated by miR-2478 from milk exosomes. Therefore, milk exosomes may be useful for the development of functional whitening cosmetics with low cytotoxicity.

## Data Availability

The datasets used and/or analyzed during the current study are available from the corresponding author on reasonable request.

## Supplementary Materials

The following are available online at https://www.mdpi.com/article/10.3390/cells10112848/s1, Figure S1: B16F10 cells were incubated with 50 μg/mL milk exosomes labeled with PKH26 (red) and were analyzed by fluorescence microscopy. Nuclei were stained with 4,6-diamidino-2-phenylindole (DAPI; blue). Figure S2: Tyrosinase activity was assessed after treatment with microRNAs.
