# Supplementary material for: Milk Exosome-Derived MicroRNA-2478 Suppresses Melanogenesis through the Akt-GSK3β Pathway
Source: Cells. 2021 Oct 22;10(11):2848. doi: 10.3390/cells10112848 (PMC8616206; doi:10.3390/cells10112848)
Supplement: Supplementary file 1 [file cells-10-02848-s001.zip › cells-1420546-supplementary.pdf]

Fig. S1

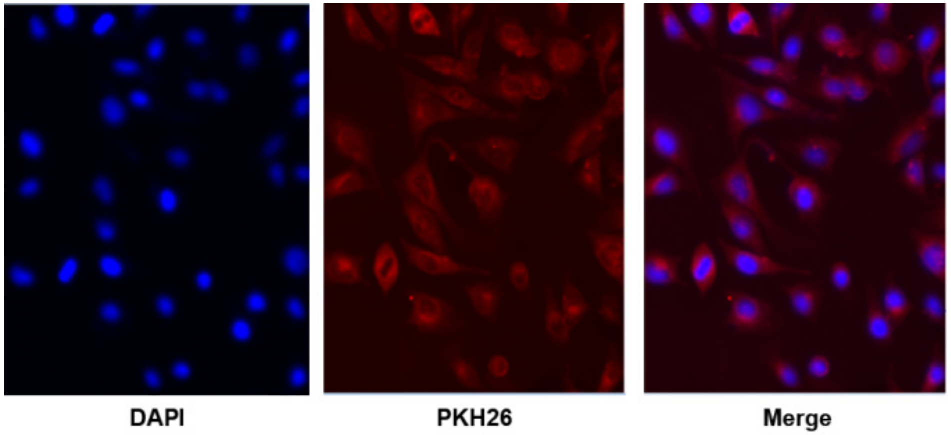

Figure S1. B16F10 cells were incubated with 50 μg/ml milk exosomes labeled with PKH26 (red) and were analyzed by fluorescence microscopy. Nuclei were stained with 4′,6-diamidino-2-phenylindole (DAPI; blue).

Fig. S2

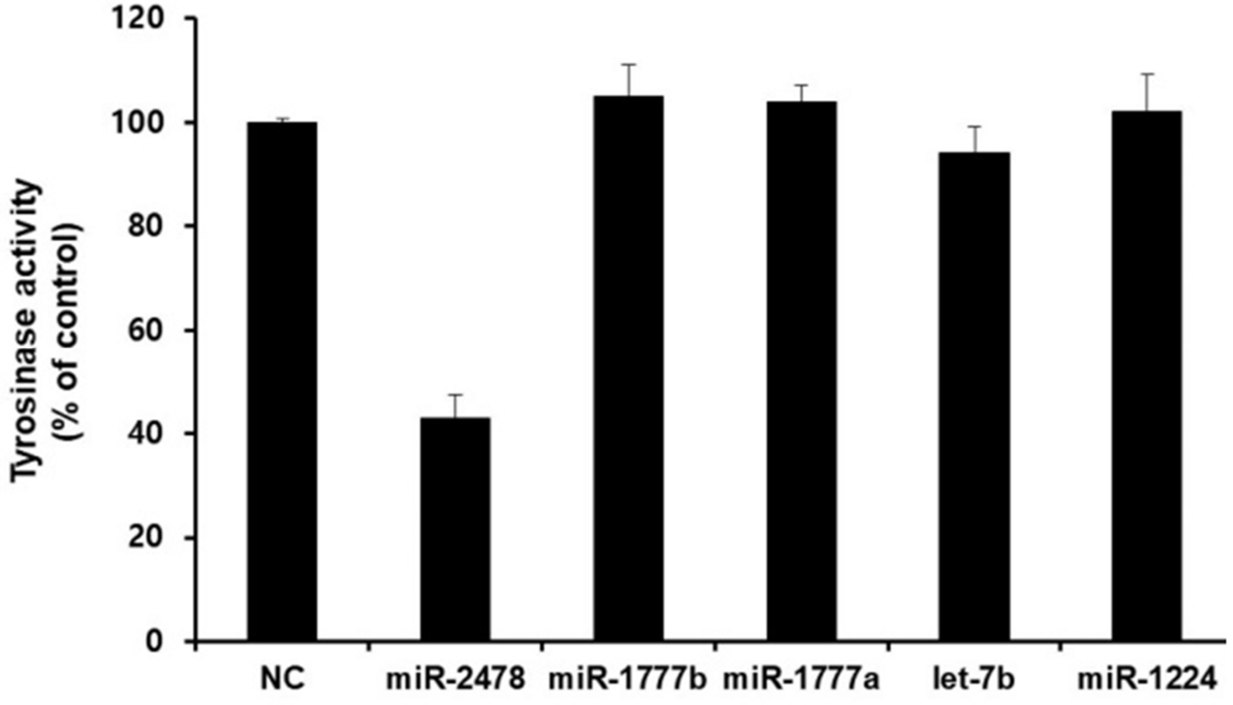

Figure S2. Tyrosinase activity was assessed after treatment with microRNAs.
